# Comparing a Model of Augmented Postpartum Primary Care to Usual Care in an Urban Medical Center

**DOI:** 10.1007/s11606-024-09165-z

**Published:** 2024-11-11

**Authors:** Sam Wainwright, Anne Elizabeth Glassgow, Abigail Holicky, Eric Kim, Melissa Wagner-Schuman, Kavya Anjur, Shreya Bellur, Rachel Caskey

**Affiliations:** 1 Division of Academic Internal Medicine, Department of Medicine, College of Medicine, University of Illinois at Chicago, Chicago, IL USA; 2 Department of Pediatrics, College of Medicine, University of Illinois at Chicago, Chicago, IL USA; 3 Department of Psychiatry, University of Illinois Chicago College of Medicine, Chicago, IL USA; 4 School of Public Health, University of Illinois at Chicago, Chicago, IL USA; 5 Department of Psychiatry and Behavioral Neuroscience, University of Cincinnati, Cincinnati, OH USA

**Keywords:** mothers, maternal health, postpartum care, fourth trimester, postpartum primary care, two-generation care

## Abstract

**Background:**

The US faces a maternal health crisis and struggles to deliver recommended postpartum care. In some populations, less than half of mothers attend a postpartum visit.

**Objective:**

To determine if a two-generation (Two-Gen) model of interdisciplinary, postpartum primary care was associated with increased visit attendance for postpartum care, primary care, and behavioral health.

**Design:**

Retrospective study of care delivered at a single, urban, academic, safety-net medical center between 2020 and 2023.

**Participants:**

Mothers who received postpartum care in Two-Gen and a comparison group who received usual postpartum care.

**Main Measures:**

Adjusted logistic regression to estimate the effect of Two-Gen participation on the odds of attending an early (birth-to-3 weeks) postpartum visit, later (4-to-12 weeks) postpartum visit, OB/GYN visit, and primary care visit.

**Key Results:**

A total of 247 mothers (98 Two-Gen and 149 usual care) were included for analysis. Most identified as Non-Hispanic Black (55%) or Hispanic (34%) and had Medicaid insurance (74%). On average, Two-Gen mothers were younger and more likely to be primiparous. Compared to usual care, Two-Gen mothers had similar rates of early postpartum visits (79% vs 64%; adjusted odds ratio (aOR) 1.70; 95% confidence interval (CI) 0.92–3.14) and were significantly more likely to have a later postpartum visit (92% vs 79%; aOR 2.46; 95%CI 1.06–5.74) in adjusted analyses. Almost all Two-Gen mothers (97%) had a visit with a primary care doctor in the first postpartum year, compared to 19% of mothers receiving usual care (aOR 12.95; 95%CI 6.80–24.68). Of those with behavioral health diagnoses, Two-Gen mothers had higher rates of psychiatrist visits than usual care mothers (49% vs 13%; *p* = 0.001).

**Conclusions:**

Two-Gen clinic participation was associated with high rates of timely postpartum care in a group of predominantly young, publicly insured, racial, and ethnic minority mothers and compared favorably to usual care across multiple metrics, notably utilization of primary and behavioral health care.

**Supplementary Information:**

The online version contains supplementary material available at 10.1007/s11606-024-09165-z.

## INTRODUCTION

The USA is facing a maternal health crisis, with unacceptably high rates of maternal mortality compared to other high-income nations.^1^ These rates are rising and becoming increasingly disparate for certain racial and ethnic groups.^2^ In Illinois, Black mothers were three times as likely to die of a pregnancy-related medical condition compared to White mothers from 2018 to 2020.^3^ Disparities in adverse maternal health outcomes are driven not only by the physical health complications of pregnancy but also by comorbidities, social determinants of health, and provider bias.^4^ Strategies to address these disparities include preconception management of risk factors and chronic conditions, and primary care visits within the first year after delivery.^5,6^ More than half of the pregnancy-related deaths in Illinois from 2018 to 2020 occurred more than 60 days postpartum, highlighting the importance of care continuity beyond the immediate postpartum period.^3^ Lastly, in Illinois, the rates of obesity, hypertension, and diabetes at delivery have all increased over the last decade (2010–2020) and many mothers experience mental health conditions and substance use during pregnancy.^3^ It is recommended that all mothers, but especially those with comorbidities, identify a primary medical home for ongoing care after pregnancy^6^; however, this integration with primary care is not well studied.

To address these adverse outcomes, the University of Illinois Health System (UI Health) established an innovative postpartum care model called the Two-Generation Clinic (Two-Gen). Two-Gen aims to deliver comprehensive, co-located postpartum primary care and family planning to mothers alongside the routine pediatric care for their infants. Two-Gen is designed to augment postpartum care in the “fourth trimester” (birth-to-12 weeks), and more tightly integrate primary and postpartum care as suggested by the American College of Obstetricians and Gynecologists (ACOG).^7^ Two-Gen integrates previous research findings that attendance at routine infant care visits is high even when the utilization of postpartum maternal visits is low.^8–10^ Two-Gen uses a collaborative care model for physical and behavioral health support utilizing a team of internal medicine-pediatrics (Med-Peds) primary care physicians, a psychiatrist, social workers, lactation specialists, and care navigators. Patients receive comprehensive screening and physical, behavioral, and social care to address the drivers of postpartum morbidity and mortality.^11^ The clinic uses multidisciplinary, shared 40-min visits and has weekly team meetings to discuss the patient panel and identify strategies to meet individual patient needs. An expanded description of the Two-Gen clinical model has been previously published.^11^ The clinic has been in operation since Fall 2020. Obstetric patients were referred to the clinic in the provision of routine care at UI Health and assignment was not randomized. The availability of augmented psychosocial support within Two-Gen was advertised within the health system.

This study evaluates the Two-Gen model after the first 2.5 years of operation to determine if there were differences in the frequency and timeliness of care for mothers in Two-Gen versus usual care within the same health system. A secondary focus was characterizing physical and behavioral health comorbidities, receipt of postpartum behavioral health care, and lactation support.

## METHODS

### Study Design

This study is a retrospective chart review comparing postpartum care (up to 12 months after delivery) received by Two-Gen mothers to mothers receiving usual postpartum care. The study is reported in alignment with the STROBE guideline for retrospective observational studies.^12^

### Setting

This study was conducted at a single, urban, academic, safety-net health system (UI Health). Care was delivered between August 2020 and March 2023. Two-Gen operates in two UI Health Med-Peds primary care practices on the South and West Sides of Chicago, Illinois. Data were abstracted from the electronic medical record (EMR) between March and July 2023, and analysis was completed in May 2024.

### Participants

All mothers enrolled in the Two-Gen clinic who were at least 3 months postpartum were included. A usual care comparison group sample was taken from all mothers who delivered at UI Health during the study period and were not enrolled in Two-Gen, had at least two prenatal visits at the UI Health Obstetrics/Gynecology (OB/GYN) clinic, and had established care before the eighth month of pregnancy. These criteria were chosen to identify mothers most likely to be well established at UI Health for their care (rather than presenting only for delivery). Mothers were excluded if they had a non-live birth, received pregnancy care in a department other than OB/GYN, were documented as receiving pregnancy care primarily outside UI Health, or moved to another geographic area. A target of two-to-one oversampling of usual care mothers was chosen to increase the chance of including women with behavioral health diagnoses given the perceived higher-than-normal presence of these comorbidities in Two-Gen mothers; a specific power calculation was not performed. Two-Gen mothers are encouraged to continue care with their obstetrician, so were not excluded for receiving care in both Two-Gen and OB/GYN. From the population of eligible mothers, 200 charts were selected by random number generation using the SAS proc surveyselect function for full chart abstraction.

Data were abstracted from the EMR for care delivered from August 2020 to March 2023 by trained abstractors who reviewed each chart individually using a standardized protocol. To ensure accuracy, a physician and study primary investigator (S.W.) reviewed all charts included in the data collection and adjudicated questions from abstractors. Data were reviewed weekly by the team’s analysts (E.K., A.H.) and questions were resolved by the PI. The study was approved by UIC’s Institutional Review Board.

### Data Collection

Socio-demographic and health characteristics were collected, including age, race/ethnicity, insurance, parity, prenatal care entry, and physical and behavioral health diagnoses. Race/ethnicity were collected from self-reported data within the EMR and were categorized as mutually exclusive groups: non-Hispanic White, non-Hispanic Black, Hispanic, non-Hispanic other, or not reported. Insurance was categorized as Medicaid, private, or other. Parity was categorized as one or more-than-one live births. Gestational age at first prenatal visit at UI Health was dichotomized as first trimester entry or not. Diagnoses were obtained from the EMR within the problem list (*International Classification of Diseases (ICD), Tenth Revision* codes) or documented in clinical notes. Similar diagnoses were combined into categories. For example, “diabetes” includes patients who have any type of diabetes including pre-existing type 2 diabetes mellitus and gestational diabetes (see Supplementary Table 1). Categories were selected based on comorbidities associated with maternal morbidity but were not exhaustive.^13–15^

Completed visits in Two-Gen, OB/GYN, or other primary care clinics at UI Health were recorded for the first postpartum year, and based on visit date were categorized as early postpartum (birth-to-3 weeks) or later postpartum (4-to-12 weeks). For the fourth trimester, primary outcome measures were dichotomized as yes/no for the following: an early postpartum visit, a later postpartum visit, at least one visit to OB/GYN, and at least one visit to primary care. A visit in OB/GYN or Two-Gen was counted as a postpartum visit. A visit in Two-Gen or other primary care clinic was counted as a primary care visit. The measures for at least one visit to OB/GYN and primary care were also calculated for the first postpartum year. Receipt of behavioral health care was measured separately, as completion of a clinical visit with a social worker/therapist, psychiatrist, or psychologist. Psychiatry referrals, documented phone calls/messages related to postpartum care, and lactation consultations were also recorded as counts. Scores on the Edinburgh Postnatal Depression Scale (EPDS) or Patient Health Questionnaire-9 (PHQ-9) were recorded, if reported. Both are used routinely in postpartum care at UI Health, according to clinic and provider preference. A positive depression screening was a score of 10 or higher and/or endorsement of suicide ideation on either the EPDS or the PHQ-9.^16^ Recorded receipt of subspecialty care was limited to mental and behavioral health care due to the link between unmet behavioral health needs and maternal mortality in Illinois and nationally.^3,17,18^

### Statistical Analysis

Statistical analysis was performed using SAS OnDemand. Descriptive and differential statistics were calculated for patient characteristics overall and by care group. For continuous data, differences between groups were calculated using independent *t*-test or Wilcoxon’s rank sum test depending on the normality of the data (Shapiro–Wilk test). For categorical data, Pearson’s Chi-square test or two-tailed Fisher’s exact test was used to test for differences.

For our primary outcome measures of postpartum care visit utilization, we first used logistic regression to calculate crude odds ratios for the association between care group and each postpartum care utilization outcome separately. We then used multivariate logistic regression to adjust for between-group differences across multiple patient characteristics. We used single-factor adjusted logistic regression to determine significant patient characteristics using a *p* < 0.05 to establish statistical significance. The final multivariate logistic regression models were adjusted for maternal age and parity, which were the only demographic factors that were significant in the single-factor models. For our secondary outcomes, we calculated unadjusted differential statistics for patients in Two-Gen versus usual care groups.

In both groups, about one-quarter of patients had less than a full year between delivery and chart abstraction, but all patients had the full fourth trimester available for review. Sensitivity analysis was performed by excluding incomplete cases from analysis to minimize bias. Exclusion did not meaningfully change the differences reported between the groups indicating the robustness of our findings. For clarity, we restricted results reported specifically for the birth-to-1-year time frame to include only the patients with at least 365 days of follow-up.

In line with UIC’s best practices for the use of race in health research, we present descriptive statistics that include “race,” which we include here as a proxy for racism.^19^ Though “race” is socially constructed and has no genetic basis, racism has real physiological, political, and economic consequences in the postpartum care of mothers. These consequences are rooted in state-sanctioned historical and contemporary racial oppression and manifest in differences in care received by mothers of different backgrounds. Race is thus reported to provide this necessary context for the care received by mothers in this study.

## RESULTS

**Figure 1:**
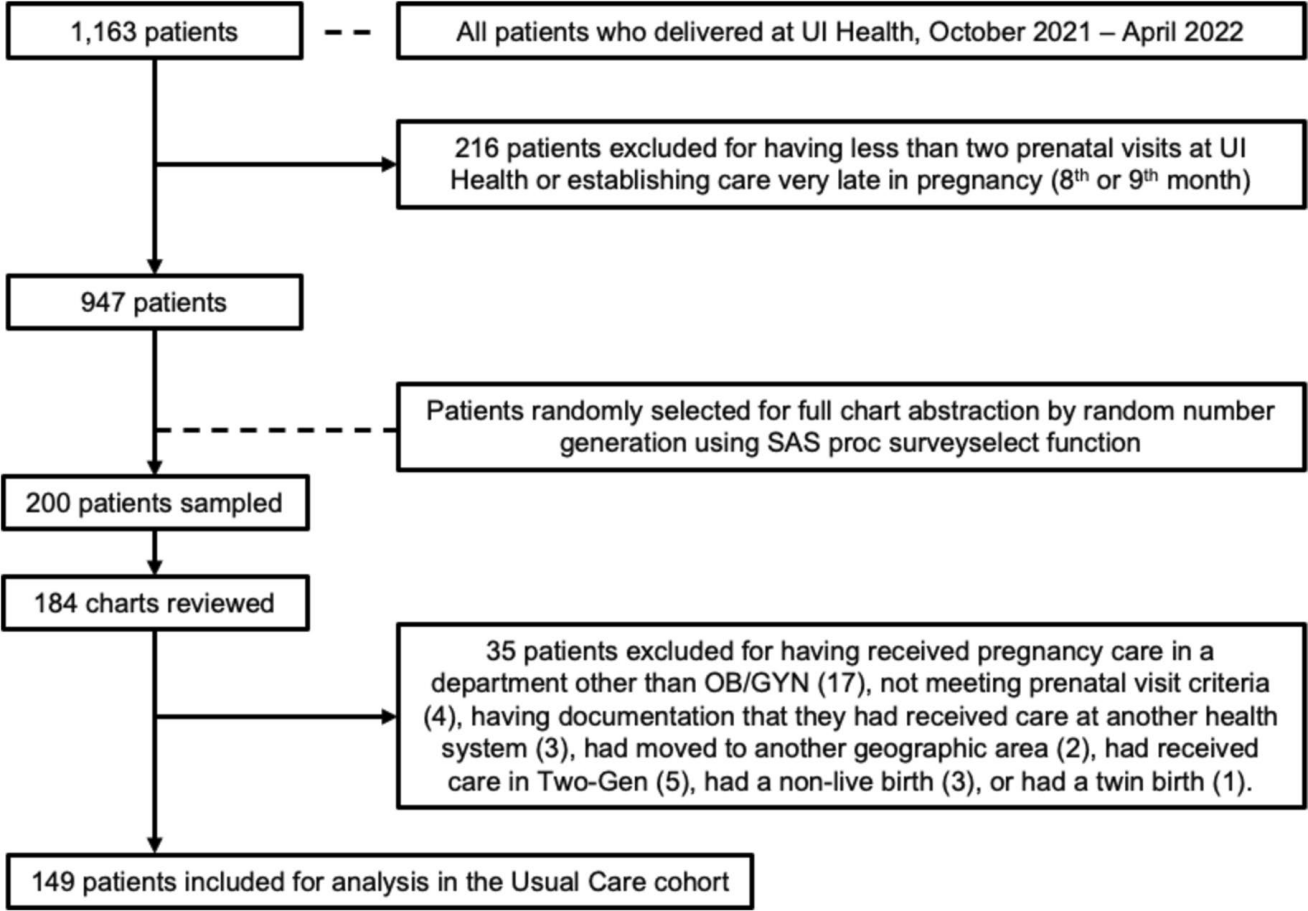
Study usual care sample selection.

From an eligible population of 947 mothers, a usual care sample of 200 was randomly selected for full chart abstraction. Of these, 184 were completed due to time and resource constraints. Thirty-five mothers were excluded based on our specific criteria. A total of 247 mothers (98 Two-Gen and 149 usual care) were included in the analysis (Fig. 1). The socio-demographic and health characteristics of the sample are detailed in Table 1. Two-Gen and usual care mothers were similar across race/ethnicity and insurance. Two-Gen mothers were on average younger (27 vs 29 years; *p* = 0.016) and more likely to be first-time mothers (60.2% vs 32.2%; *p* < 0.001). First trimester entry into prenatal care was not significantly different between the groups. Both groups had a high prevalence of physical health diagnoses, with hypertension and obesity being the most common. Two-Gen mothers were substantially more likely to have a behavioral health diagnosis than usual care (58% vs 20%; *p* < 0.001) and almost half of Two-Gen mothers had a mood disorder diagnosis (45% vs 15%; *p* < 0.001).

**Table 1:** Socio-demographic and Health Characteristics of Patients Receiving Postpartum Care in the Two-Generation Clinic Versus Usual Care at UI Health

	Patients, no. (%)			
	Total (*n* = 247)	Two-Gen (*n* = 98)	Usual Care (*n* = 149)	*p* value
Age, mean (SD), years	28.1 (6.1)	26.9 (6.2)	28.9 (5.9)	0.016
Racial and ethnic group^a^				
Hispanic	83 (33.6)	34 (34.7)	49 (32.9)	0.86
Non-Hispanic Black	135 (54.7)	52 (53.1)	83 (55.7)	
Non-Hispanic White	13 (5.3)	4 (4.1)	9 (6.0)	
Non-Hispanic Other	10 (4.1)	5 (5.1)	5 (3.4)	
Not reported	6 (2.4)	3 (3.1)	3 (2.0)	
Health insurance^a^				
Medicaid	182 (73.7)	69 (70.4)	113 (75.8)	0.39
Private	11 (4.5)	24 (24.5)	30 (20.1)	
Other/none^b^	54 (21.9)	5 (5.1)	6 (4.0)	
Parity				
1	107 (43.3)	59 (60.2)	48 (32.2)	< 0.001
> 1	140 (56.7)	39 (39.8)	101 (67.8)	
First trimester entry into prenatal care	133 (53.9)	49 (50.0)	84 (56.38)	0.33
Physical health diagnosis^c^	202 (81.8)	78 (79.6)	124 (83.2)	0.47
Asthma	58 (23.5)	24 (24.5)	34 (22.8)	0.76
Diabetes	45 (18.2)	13 (13.3)	32 (21.5)	0.10
Hypertension	104 (42.1)	41 (41.8)	63 (42.3)	0.95
Obesity	104 (42.1)	34 (34.7)	70 (47.0)	0.06
Pre-eclampsia or eclampsia	71 (28.7)	28 (28.6)	43 (28.9)	0.96
Behavioral health diagnosis^d^	87 (35.2)	57 (58.2)	30 (20.1)	< 0.001
Mood disorders	68 (27.5)	45 (45.9)	23 (15.4)	< 0.001
Anxiety disorders	46 (18.6)	34 (34.7)	12 (8.1)	< 0.001
Post-traumatic stress disorder	13 (5.3)	9 (9.2)	4 (2.7)	0.03
Bipolar disorders	7 (2.8)	4 (4.1)	3 (2.0)	0.44

*Abbreviations*: *SD*, standard deviation; *Two-Gen*, Two-Generation Clinic

^a^Percentages may not add up to 100% due to rounding; mutually exclusive groups

^b^Includes Student Health plans, Veterans Affairs health care, and self-pay/uninsured

^c^Includes one or more of the following physical health diagnoses: asthma, diabetes (including gestational diabetes), hypertension (including gestational hypertension), obesity (pre-gravid BMI > 30), and pre-eclampsia or eclampsia; individuals may have had more than one physical health diagnosis

^d^Includes one or more of the following behavioral health diagnoses: mood disorders (including postpartum depression), anxiety disorders, post-traumatic stress disorder, and bipolar disorders; individuals may have had more than one behavioral health diagnosis

Table 2 describes the differences in postpartum care utilization between the groups and provides crude and adjusted odds ratios, adjusted for maternal age and parity. During the fourth trimester, the likelihood of having an early postpartum visit was similar between Two-Gen and usual care mothers (78% vs 63%, adjusted odds ratio (aOR) 1.70; 95% confidence interval (CI) 0.92–3.14). Two-Gen mothers had 2.5 times higher odds of having a later postpartum visit (92% vs 79%, aOR 2.46; 95%CI 1.06–5.74) when compared to usual care. During the fourth trimester, Two-Gen mothers were less likely to have attended at least one OB/GYN visit (75% vs 88%; aOR 0.28; 95%CI 0.14–0.59). Two-Gen mothers were substantially more likely to have attended at least one primary care visit during the fourth trimester (79% vs 4%; aOR 89.7; 95%CI 33.42–240.82).

**Table 2:** Frequency, Crude, and Adjusted Odds of Receipt of Postpartum Care, Primary Care, and Obstetric Visits for Patients Receiving Postpartum Care in the Two Generation Clinic Versus Usual Care at UI Health

	Patients, mo. (%)				
	Total	Two-Gen	Usual care	Crude odds ratio (95% CI)	Adjusted odds ratio^a^ (95% CI)
Fourth trimester (birth-to-12 weeks)	*n* = 247	*n* = 98	*n* = 149		
Early postpartum visit^b^	172 (69.6)	77 (78.6)	95 (63.8)	2.08 (1.16–3.75)	1.70 (0.92–3.14)
Later postpartum visit^c^	207 (83.8)	90 (91.8)	117 (78.5)	3.08 (1.35–7.00)	2.46 (1.06–5.74)
At least 1 visit to obstetrics	204 (82.6)	73 (74.5)	131 (87.9)	0.35 (0.18–0.70)	0.28 (0.14–0.59)
At least 1 visit to primary care	83 (33.6)	77 (78.6)	6 (4.0)	87.39 (33.84–225.66)	89.71 (33.42–240.82)
First postpartum year (birth-to-1 year)^d^	*n* = 186	*n* = 71	*n* = 115		
At least 1 visit to obstetrics	159 (85.5)	55 (77.5)	104 (90.4)	0.55 (0.33–0.94)	0.48 (0.28–0.85)
At least 1 visit to primary care	91 (48.9)	69 (97.2)	22 (19.1)	13.73 (7.34–25.70)	12.95 (6.80–24.68)

*Abbreviations*: *CI*, confidence interval; *OB/GYN*, obstetrics/gynecology; *SD*, standard deviation; *Two-Gen*, Two-Generation Clinic

^a^Results from logistic regression model with Wald’s test. Model adjusted for maternal age at delivery and parity

^b^Defined as at least one visit within 0-to-3 weeks postpartum with a primary care or obstetrics provider

^c^Defined as at least one visit within 4-to-12 weeks postpartum with a primary care or obstetrics provider

^d^Analysis restricted to mothers for whom at least 365 days of follow-up time was available for analysis (Two-Gen *n* = 71 (72% of full group), usual care *n* = 115 (77% of full group))

For birth-to-1-year measures, analysis was restricted to mothers for whom at least 365 days of follow-up time was available for analysis. Almost all Two-Gen mothers (97%) had a visit with a primary care doctor in the first postpartum year, compared to 19% of mothers receiving usual care (aOR 12.95; 95%CI 6.80–24.68). In total, 77% of Two-Gen mothers had at least one OB/GYN visit in the first year compared to 90% of mothers receiving usual care (aOR 0.48; 95%CI 0.28–0.85).

Table 3 characterizes additional postpartum services received by the two groups. Two-Gen mothers had fewer OB/GYN visits on average compared to usual care (1.8 vs 2.6 visits; *p* = 0.002), but significantly more primary care visits (5.9 vs 0.4 visits; *p* < 0.001). Notably, Two-Gen mothers received significantly more phone calls and messages related to postpartum care, averaging 14.6 phone calls or messages compared to 1.6 in usual care (*p* < 0.001). Two-Gen mothers were more likely to have documented consultation with a lactation specialist (67% vs 15%; *p* < 0.001). Half of all Two-Gen patients received at least one therapy/counseling session with a mental health professional, compared to 6% of patients receiving usual care (*p* < 0.001). Of those with a behavioral health diagnosis, 49% of Two-Gen mothers had a visit with a psychiatrist compared to only 13% of usual care mothers (*p* = 0.006). For mothers with a positive depression screen, 50% of those in Two-Gen were seen by a psychiatrist versus 13% of those in usual care (*p* = 0.01).

**Table 3:** Additional Postpartum Clinical Services Received over the First Postpartum Year, Two-Generation Clinic Versus Usual Care at UI Health

	Patients, no. (%)		
	Two-Gen (*n* = 98)	Usual care (*n* = 149)	*p* value
Total clinic visits to OB/GYN, mean (SD) [Min, Max]	1.8 (1.5) [0.0,7.0]	2.6 (1.7) [0.0,8.0]	0.002
Total clinic visits to primary care, mean (SD) [Min, Max]	5.9 (3.5) [0.0,17.0]	0.4 (0.9) [0.0,5.0]	< 0.001
Phone calls/messages, mean (SD) [Min, Max]	14.6 (10.6) [0.0,47.0]	1.6 (4.0) [0.0,41.0]	< 0.001
Lactation consultation	66 (67.3)	23 (15.4)	< 0.001
Depression screening completed^a^	91 (92.8)	127 (85.2)	0.069
Therapy session	50 (51.0)	9 (6.0)	< 0.001
Of those with behavioral health diagnoses^b^	*n* = 57 (58%)	*n* = 30 (20%)	< 0.001
Psychiatry referral	24 (42.1)	4 (13.3)	0.006
Psychiatry visit completed	28 (49.1)	4 (13.3)	0.001
Of those with positive depression screening^c^	*n* = 36 (37%)	*n* = 16 (10%)	< 0.001
Psychiatry referral	17 (47.2)	3 (18.8)	0.05
Psychiatry visit completed	18 (50.0)	2 (12.5)	0.01

*Abbreviations*: *SD*, standard deviation; *Two-Gen*, Two-Generation Clinic; *EPDS*, Edinburgh Postnatal Depression Scale; *PHQ-9*, the Patient Health Questionnaire-9

^a^Documentation of at least one completed EPDS or PHQ-9 screening instrument during a clinical visit

^b^Includes one or more of the following behavioral health diagnoses: mood disorders (including postpartum depression), anxiety disorders, post-traumatic stress disorder, and bipolar disorders

^c^Score of ≥ 10 or suicide ideation as indicated using the EPDS or PHQ-9

## DISCUSSION

Mothers receiving care in Two-Gen had high rates of timely postpartum care visits, including a significantly higher rate of later postpartum care visits than mothers receiving usual care, even when adjusting for maternal age and parity. Care in Two-Gen was associated with lower rates of visits to OB/GYN but dramatically higher rates of visits to primary care, and with a higher number of overall clinic visits in first postpartum year. Two-Gen mothers utilized more care in the fourth trimester despite being more likely to be younger, first-time mothers, and being much more likely to have a mental health diagnosis, all of which have been previously identified as barriers to receiving postpartum care.^8,20^ These results highlight that the comprehensive, integrated nature of the Two-Gen model can increase mothers’ access to necessary physical and mental health support postpartum.

Appraising these results requires acknowledging the striking prevalence of mental health diagnoses in the Two-Gen mothers who, despite this, were more likely than a sample of mothers receiving usual care to have achieved most markers of timely postpartum care utilization examined. Over a third of Two-Gen mothers had a positive depression screening during the first postpartum year, more than double the rate of those receiving usual care. We suspect the high rate of behavioral health needs among Two-Gen mothers reflects that such patients were disproportionately referred to Two-Gen due to the promoted availability of behavioral health care within the clinic. There is also the possibility of detection bias, as Two-Gen’s expressed focus on increasing mental health screening and support for new mothers may systematically increase detection of these conditions compared to usual care. Our model is in contrast to the shortage of mental health care professionals and limited access to timely care impacting patients in Illinois and nationally, particularly for those who are publicly insured.^21–23^ Compared to usual care, Two-Gen connected more mothers with identified behavioral health needs to support (therapy or psychiatry). We believe the efforts of the wraparound team (social workers and health coaches) in reaching out to patients were a key component in achieving high rates of recommended postpartum care utilization. The average of 14.6 EMR-documented calls and messages for Two-Gen mothers is a dramatic difference from usual care, even when accounting for various clinics’ practices for documenting phone interactions.

We are not surprised to discover that only one-in-five women receiving usual care had documentation of a visit with a primary care doctor within the postpartum year. A recent study by Gemkow et al. found that among almost 8000 women receiving care at a national network of Federally Qualified Health Centers (FQHCs), only 17% of women attended a primary care visit in the first 6 months postpartum.^24^ In addition, many young women may utilize their OB/GYN clinician for primary care.^25,26^ Connection to comprehensive primary care during the postpartum period is critical for women at risk for adverse outcomes to provide management of chronic disease.^27^ The chronic physical and behavioral health conditions that contribute to postpartum morbidity and mortality risk such as diabetes, hypertension, substance use, or depression all benefit from ongoing primary health care and are not overtly obstetric in nature.^28,29^ The slightly lower rate of OB/GYN visits among Two-Gen mothers is balanced by the dramatically higher rate of primary care visits and may reflect that many postpartum care needs such as managing acute and chronic disease, screening for mental health needs, and providing contraception are within the scope of primary care.^6,30^ It may be that Two-Gen’s wraparound supports and frequent contact increased attendance in primary care but not in OB/GYN, that Two-Gen mothers substituted follow-up with Two-Gen for OB/GYN visits, or potentially that patients with an a *priori* lower likelihood of attending postpartum follow-up were referred to Two-Gen.

This study has many limitations. It is a retrospective review from a single health system and misses care occurring outside UI Health. We believe our usual care comparison group findings are a reasonable approximation of typical postpartum care patterns as they resemble the limited data reported elsewhere.^24,31–33^ We specifically sampled comparison mothers who received regular prenatal care at UI Health to exclude mothers who came only at the time of delivery. We think this identifies mothers more likely to receive their routine health care (including primary care) at UI Health and attempts to minimize ascertainment bias. We cannot be certain that usual care mothers were not receiving primary or obstetrical care elsewhere. Our usual care sample excluded mothers receiving maternity care through family medicine, and thus does not include mothers receiving both obstetric and primary care by family medicine. The vast majority of US maternity care is provided by OB/GYN physicians, with less than 2% of family physicians practicing high-volume obstetrical care; thus, we believe our usual care group best mirrors typical postpartum care.^34,35^ Our study does not directly address whether Two-Gen’s model of postpartum primary care could be generalized to other primary care clinics with similar results. In our study, most women receiving usual care did not see primary care at all despite an equally high prevalence of physical health comorbidities, suggesting a significant gap that must be filled by a broad range of primary care providers. Our experience delivering this care suggests that a key component is consistent and early postpartum engagement with mothers on their whole-person health, but further study is needed.

## CONCLUSION

Addressing the maternal health crisis requires implementing new clinical models to deliver timely and comprehensive postpartum care to identify and address the physical, behavioral, and social care needs of mothers. Receiving care in a dedicated postpartum primary care clinic was associated with high rates of timely postpartum care in a group of predominantly young, racial, and ethnic minority mothers. The model’s collaborative care and intergenerational approach potentially contributed to higher-than-expected delivery of both primary and behavioral health care services across the postpartum year compared to mothers receiving usual care. The high comorbidity burden in both groups emphasizes the importance of connecting mothers to longitudinal primary care. Though the intervention was not randomized, its apparent benefit in retrospective analysis suggests the model’s potential to improve mothers’ uptake of postpartum care and may be a strong strategy to mitigate disparities and promote health equity.

## Supplementary Information

Below is the link to the electronic supplementary material.

Supplementary file1 (DOCX 17.0 KB)

## Data Availability

The dataset generated and analyzed for this study is not publicly available due to patient privacy but is available from the corresponding author on reasonable request.

## Abbreviations

ACOG: American College of Obstetricians and Gynecologists
EMR: Electronic medical record
EPDS: Edinburgh Postnatal Depression Scale
ICD-10: International Classification of Diseases, Tenth Revision
Med-Peds: Internal medicine – pediatrics
OB/GYN: Obstetrics/gynecology
PHQ-9: Patient Health Questionnaire-9
Two-Gen: UI Health Two-Generation Clinic
UIC: University of Illinois at Chicago
UI Health: University of Illinois Hospital and Health Sciences System
