# Supplementary material for: Comparing a Model of Augmented Postpartum Primary Care to Usual Care in an Urban Medical Center
Source: J Gen Intern Med. 2024 Nov 11;40(4):854–61. doi: 10.1007/s11606-024-09165-z (PMC11914437; doi:10.1007/s11606-024-09165-z)
Supplement: Supplementary file 1 — Supplementary file1 (DOCX 17.0 KB) [file 11606_2024_9165_MOESM1_ESM.docx]

**Supplemental Appendix:**

**Supplementary Table 1: Comorbidity Categories, Included Diagnoses and Corresponding ICD-10 Codes**

| **Category** | **Included Diagnoses** |
| --- | --- |
|  | **Physical Health Comorbidities** |
| Asthma | Asthma (J45.xxx),  Diseases of the respiratory system complicating pregnancy (O99.5-O99.519) |
| Diabetes | Type 1 Diabetes Mellitus (E10.xxxx),  Type 2 Diabetes Mellitus (E11.xxxx),  Diabetes in pregnancy, including gestational diabetes (O24.xxx) |
| Hypertension | Essential Hypertension (I10.xx),  Pre-existing hypertension complicating pregnancy, childbirth and the puerperium (O10.xxx),  Pre-existing hypertension with pre-eclampsia (O11.x)^a^,  Gestational [pregnancy-induced] hypertension without significant proteinuria (O13.xx),  Unspecified maternal hypertension (O16.x) |
| Obesity | Calculated from numerical value recorded for Pre-Gravid BMI in EMR’s pregnancy episode; patients categorized as having obesity if Pre-Gravid BMI ≥ 30. |
| Pre-Eclampsia or Eclampsia | Pre-existing hypertension with pre-eclampsia (O11.x)^a^  Pre-eclampsia (O14.xx)  Eclampsia (O15.x) |
|  | **Behavioral Health Comorbidities** |
| Mood Disorders | Major Depressive Disorder (F33.xx),  Postpartum Depression (F53.0),  Depression, unspecified (F32.A) |
| Anxiety Disorders | Generalized Anxiety Disorder (F41.1),  Anxiety disorder, unspecified (F41.9),  Panic Disorder (F41.0) |
| Post-traumatic Stress Disorder | Post-traumatic stress disorder (F43.1) |
| Bipolar Disorders | Bipolar I Disorder (F31.xx),  Bipolar II Disorder (F30.xx) |

Abbreviations: BMI, Body-Mass Index; EMR, Electronic Medical Record; ICD-10, the International Classification of Disease, 10th Revision.

^a^ Pre-existing hypertension with pre-eclampsia (O11.x) is included in both the Hypertension and the Pre-eclampsia or Eclampsia diagnosis categories.
